# Biofunctionalization of Stem Cell Scaffold for Osteogenesis and Bone Regeneration

**DOI:** 10.3390/biom15121700

**Published:** 2025-12-05

**Authors:** Qianqian Chen, Yixue Jiang, Xiaoyuan Zhang, Shichun Xu, Yunwei Wang, Dan Xing, Hui Li

**Affiliations:** 1 Third Affiliated Hospital of Zhejiang Chinese Medical University, Hangzhou 310005, China; 202311110811036@zcmu.edu.cn (Q.C.); 202411110811039@zcmu.edu.cn (Y.W.); 2 Arthritis Clinic & Research Center, Zhejiang Chinese Medical University, Hangzhou 310053, China; 3 Arthritis Clinic & Research Center, Peking University People’s Hospital, Peking University, Beijing 100044, China; 1810301240@pku.edu.cn (X.Z.); 2511210286@stu.pku.edu.cn (S.X.); 4 Arthritis Institute, Peking University, Beijing 100044, China; 2410301256@stu.pku.edu.cn

**Keywords:** biofunctionalization, stem cell, bone regeneration, scaffold, visualization research

## Abstract

Repairing bone defects resulting from trauma, tumor resection, or pathological conditions remains a significant clinical challenge. While conventional bone grafting techniques are constrained by inherent limitations, stem cell-based tissue engineering scaffolds offer a promising therapeutic alternative. The ideal scaffold should not only serve as a structural support but, more importantly, must establish a bioactive microenvironment capable of directing stem cell osteogenic differentiation. Consequently, scaffold biofunctionalization has emerged as a fundamental strategy for achieving effective bone regeneration. This review comprehensively examines the primary materials utilized in bone regeneration scaffolds and systematically analyzes key biofunctionalization approaches, including surface modification, incorporation of bioactive molecules, and integration of functional ions. Moreover, it thoroughly discusses the mechanisms through which biofunctionalized scaffolds modulate stem cell behavior and enhance bone repair in vivo. Finally, the article provides insights into future research directions and emerging trends, aiming to inform the rational design and development of advanced bone regeneration scaffolds.

## 1. Introduction

Bone is a mineralized connective tissue with remarkable regenerative capacity and plays multiple vital roles, including mechanical support, protection of internal organs, facilitation of movement, mineral storage, and housing of bone marrow [1,2]. However, bone repair remains a significant challenge, particularly in cases involving large defects, osteoporosis, or post-traumatic injury. In recent years, mesenchymal stem cells (MSCs) have emerged as a key cell source for bone tissue regeneration due to their multipotent differentiation capacity and immunomodulatory properties [3]. Successful bone regeneration, however, depends not only on the intrinsic potential of MSCs but also on an appropriate scaffold that provides both mechanical support and a conducive microenvironment for osteogenesis.

Traditional single-component bone scaffolds offer basic mechanical stability but exhibit limited bioactivity and suboptimal interaction with MSCs. Moreover, the complex bone-healing microenvironment—characterized by inflammation, hypoxia, and poor vascularization—often compromises stem cell survival and differentiation, resulting in incomplete regeneration [4]. These challenges have driven increasing interest in functionalized scaffold systems designed to enhance MSC survival and osteogenic efficiency under pathological conditions.

Biofunctionalized scaffolds integrate bioactive coatings, controlled release systems, and environment-responsive features to improve cell adhesion, proliferation, and lineage commitment [5]. Such scaffolds can recruit endogenous stem cells, modulate inflammatory responses, and promote angiogenesis, thereby supporting coordinated bone repair [6]. Through these advances, scaffold functionalization has become a research focus in bone tissue engineering, bridging the gap between basic research and clinical translation [7].

Despite rapid progress, comprehensive reviews integrating both experimental findings and bibliometric insights into this field remain limited. Functionalization has also become a prominent theme in regenerative medicine, and bibliometric approaches provide valuable tools for predicting innovation trends and emerging directions. Therefore, this review systematically summarizes progress in biofunctionalized stem cell scaffolds for bone defect repair, incorporating studies published between 2014 and 2024. By combining literature synthesis with bibliometric analysis, we aim to provide a comprehensive overview of current advances and offer perspectives for future development in the rational design of biofunctionalized scaffolds for bone regeneration.

## 2. Bone Regeneration

### 2.1. The Physiological Process of Bone Regeneration

Bone is a mineralized connective tissue with a high intrinsic capacity for self-repair. The healing process generally proceeds through three overlapping stages: inflammation, repair, and remodeling [8,9]. During the initial inflammatory phase, immune and inflammatory cells infiltrate the injury site, clear necrotic tissue, and release chemokines that recruit mesenchymal stem cells (MSCs) [10,11,12,13]. Depending on the injury site and microenvironmental conditions, the repair phase can occur through either endochondral or intramembranous ossification [14]. Osteoprogenitor cells differentiate into osteoblasts that secrete the osteoid matrix, while osteoclasts participate in bone resorption and remodeling. These processes gradually restore the structural and mechanical integrity of the tissue [15,16]. Osteocytes—terminally differentiated osteoblasts embedded within the bone matrix—serve as mechanosensors that regulate bone homeostasis by secreting signaling molecules such as sclerostin and RANKL [17,18]. Through this regulatory network, osteocytes coordinate bone formation and resorption, maintaining the balance necessary for skeletal remodeling and repair.

### 2.2. Microenvironmental Characteristics of Bone Regeneration

Bone regeneration depends on a highly dynamic local microenvironment comprising inflammatory mediators, immune cells, angiogenic factors, and mechanical cues. The inflammatory response is essential for the early clearance of necrotic tissue and stem cell recruitment, but excessive or prolonged inflammation can impair osteogenesis [19,20]. Furthermore, the regeneration of the vascular-nerve network is crucial for bone regeneration, as it not only provides oxygen and nutrients but also participates in the regulation of osteogenic signals through the secretion of neurotrophic factors [10,21]. In addition, the mechanical properties of the extracellular matrix—such as stiffness and topography—directly influence stem cell fate decisions through mechanotransduction pathways [22]. Therefore, bone regeneration is governed by a highly coordinated interplay of biochemical and biomechanical cues that must be precisely regulated in space and time.

### 2.3. Pathological Microenvironment Inhibition of Regeneration

Under pathological conditions such as diabetes mellitus or chronic inflammation, bone regeneration is markedly impaired. Elevated oxidative stress and increased matrix metalloproteinase (MMP) activity disrupt stem cell homeostasis, thereby inhibiting osteogenic differentiation and bone matrix formation [23,24]. Moreover, replicating the complex hierarchical architecture of native bone—particularly the gradual mineralization pattern within Haversian systems—remains difficult with conventional scaffolds, resulting in incomplete restoration of mechanical integrity [24]. Delayed angiogenesis and neural network reconstruction further contribute to suboptimal osseointegration and delayed healing [10,21].

Consequently, a major challenge in current bone tissue engineering lies in how to effectively reconstruct or modulate the pathological microenvironment to restore osteogenic activity and re-establish the functional coupling of vascularization, innervation, and bone formation.

## 3. Stem Cell

Although bone possesses a remarkable capacity for self-repair, this intrinsic potential is insufficient for large or complex defects—such as those exceeding 2–3 cm in length—or in pathological conditions like chronic osteoarthritis, where the regenerative capacity of cartilage and bone is compromised. These limitations have stimulated the development of novel regenerative strategies, with stem cell–based therapy emerging as one of the most promising approaches for bone repair and regeneration.

### 3.1. Classification and Characteristics of Stem Cells

Stem cells are undifferentiated cells characterized by their self-renewal ability and multipotent differentiation capacity. While stem cells from different tissue sources may exhibit similar morphology and immunophenotype, their proliferation capacity and differentiation potential vary significantly. Among these, mesenchymal stem cells (MSCs) are the principal cell type used in bone regeneration due to their multilineage differentiation capability, high proliferative potential, and low immunogenicity [25,26]. Based on their origin, MSCs can be classified into several types, including bone marrow–derived MSCs (BM-MSCs), adipose-derived stem cells (ADSCs), dental pulp stem cells (DPSCs), and umbilical cord–derived MSCs (UC-MSCs). These cells can differentiate into osteoblasts, chondrocytes, and adipocytes, among others, enabling their use in various orthopedic and dental applications. Among them, BM-MSCs are the most extensively studied and have shown great potential in the treatment of spinal, joint, and osteoporotic disorders [27]. As non-hematopoietic stem cells, BM-MSCs contribute to skeletal development and maintenance primarily through differentiation into osteoblasts and other bone-lineage cells. In addition to adult stem cells, induced pluripotent stem cells (iPSCs) have gained increasing attention in bone regeneration research [26,28]. iPSCs possess self-renewal capacity and the potential to differentiate into virtually any somatic cell type, offering an unlimited source of patient-specific stem cells. However, their clinical application still requires careful evaluation of safety issues such as tumorigenicity and genomic instability.

### 3.2. Mechanisms of Stem Cell-Promoted Osteogenesis

The mechanisms by which stem cells promote bone regeneration are multifactorial, involving direct differentiation, paracrine signaling, and immunomodulation. Among these, the most direct mechanism is osteogenic differentiation, through which stem cells transform into bone-forming cells that synthesize bone matrix and facilitate mineralization.

During this process, osteoblasts produce key extracellular matrix components such as type I collagen and osteocalcin, followed by hydroxyapatite deposition that forms the mineralized bone matrix. These events are tightly regulated by multiple signaling pathways, among which the bone morphogenetic protein (BMP) and Wnt/β-catenin pathways are particularly important. BMP signaling activates specific receptor-regulated Smads (R-Smads), namely *Smad1*, *Smad5*, and *Smad8*. These activated proteins bind with the common-mediator Smad (Co-Smad) Smad4 to form a complex. This complex subsequently translocates into the nucleus, where it upregulates the transcription factor *Runx2*, a master regulator of osteogenesis [29,30,31,32]. Meanwhile, activation of the Wnt/β-catenin pathway promotes osteoblastic differentiation while suppressing adipogenic lineage commitment [33,34].

Beyond direct differentiation, stem cells facilitate bone repair through paracrine effects, primarily by secreting bioactive molecules that modulate the local microenvironment. Among these secreted factors, extracellular vesicles (EVs)—especially exosomes—act as essential mediators of intercellular communication. These vesicles carry proteins, mRNA, and miRNA that can influence the proliferation, migration, and differentiation of surrounding cells [35]. In addition, stem cells release proangiogenic factors such as vascular endothelial growth factor (VEGF) and angiopoietin-1 (Ang-1), which stimulate neovascularization at the defect site, ensuring sufficient oxygen and nutrient supply for regenerating bone tissue [36]. Such paracrine signaling also enhances the recruitment and osteogenic differentiation of endogenous progenitor cells, thereby amplifying the regenerative response [37].

The interaction between stem cells and the immune system is another key mechanism underlying successful bone repair. In the early inflammatory phase of bone repair, stem cells secrete multiple anti-inflammatory factors such as interleukin-10 (IL-10) and transforming growth factor-β (TGF-β). These factors are involved in immune regulation by modulating the behavior of immune cells, particularly the function of macrophages and lymphocytes. They suppress excessive inflammatory responses while avoiding inhibition of osteogenic activity, resulting in a favorable microenvironment for tissue repair [19].

**Figure 1:**
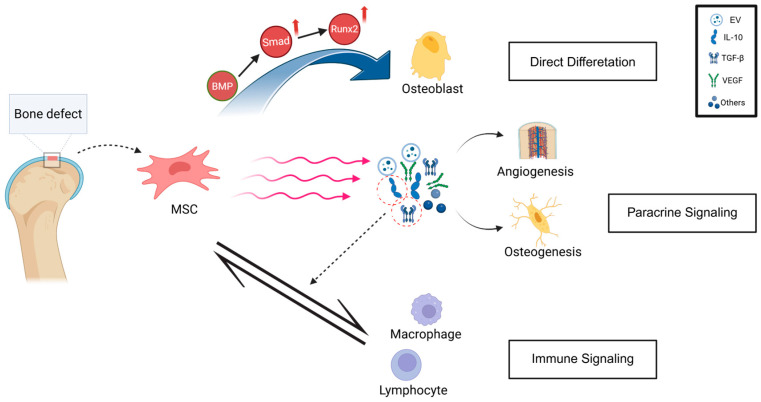
Mechanisms of MSC-mediated bone regeneration. MSCs promote bone repair through osteogenic differentiation, paracrine secretion of bioactive factors, and immunomodulation that will be beneficial for regeneration. Created with BioRender.com.

In summary, stem cells promote bone regeneration through three interrelated mechanisms: (1) direct differentiation into osteogenic cells, (2) paracrine signaling that recruits and activates surrounding cells, and (3) immunomodulation that shapes a microenvironment favorable for tissue repair (Figure 1).

### 3.3. Limitations

Despite its remarkable promise in bone repair, stem cell–based therapy still faces several critical limitations. Low post-transplantation survival rates and functional deterioration are among the major obstacles. In patients with osteoarthritis, for instance, the inflammatory microenvironment impairs mesenchymal stem cell (MSC) migration, proliferation, and differentiation, while hypoxia and ischemia accelerate cellular senescence [38]. Yu et al. reported apoptosis of allogeneic MSCs transplanted into rat bone defect models and observed that apoptosis inhibition paradoxically suppressed bone regeneration. Similarly, Fahy et al. found that conditioned medium derived from osteoarthritic synovium significantly reduced MSC viability, secretion of trophic factors, and chondrogenic potential [39].

In addition to survival issues, both the efficacy and safety of stem cell therapy require rigorous evaluation. Therapeutic outcomes depend on multiple variables, including cell source, patient condition, and delivery strategy. Most current studies remain at the preclinical stage, highlighting the need for well-designed human trials to establish clinical efficacy. Another unresolved issue is the lack of standardized quality control for stem cell preparations. Autologous transplantation often involves invasive harvesting procedures, and cell yield and quality may vary with patient age and health status [28,40]. Allogeneic transplantation, while more practical, carries risks of pathogen transmission and immune rejection. Although immunosuppressive regimens can mitigate rejection, they inevitably increase susceptibility to infection and other complications.

Furthermore, while induced pluripotent stem cells (iPSCs) provide an unlimited patient-specific source, their use still raises safety concerns, including tumorigenicity and genomic instability [28]. In summary, although stem cells hold great potential for bone defect repair, challenges related to survival, safety, reproducibility, and standardization must be addressed before these therapies can achieve reliable clinical translation.

## 4. Stem Cell Scaffold for Osteogenesis and Bone Regeneration

### 4.1. Properties of Scaffold Materials

An ideal biomaterial for bone regeneration should exhibit biochemical and physical properties to ensure biocompatibility, bioactivity, and mechanical integrity in vivo [41,42,43].

#### 4.1.1. Biochemical Properties

In bone tissue engineering, the biochemical properties of scaffold materials are essential factors determining their ability to successfully guide tissue regeneration. These properties collectively define the complex interactions at the material-biological interface and regulate the biologic response of the hospital from the initial moment of implantation. They primarily encompass three interrelated aspects: biocompatibility, bioactivity, and degradability.

Firstly, biocompatibility is the most fundamental and essential requirement for any biological scaffold. After implantation, the scaffold material should not trigger severe inflammatory responses or immune rejection and must remain non-toxic to surrounding cells and tissues [44,45]. The surface of materials—including its wettability, charge, and functional groups—constitutes the primary point of contact with bodily fluids and cells. It directly determines the type, quantity, and conformation of adsorbed key proteins such as fibronectin. This adsorbed protein layer subsequently regulates stem cell adhesion, spreading, and differentiation through integrin-mediated signaling pathways. Therefore, surface modification of biomaterials is crucial for regulating cell behavior [46,47,48]. For illustration, scaffold materials bearing polar functional groups such as carboxyl or amino groups significantly enhance the ordered adsorption of fibronectin and the exposure of its RGD sequence. This, in turn, amplifies α5β1 integrin-mediated signaling, thereby promoting the osteogenic differentiation of mesenchymal stem cells [49].

Secondly, an ideal scaffold should possess chemically biologically active properties. Chemically biologically active properties refer to materials that do not merely remain inert but actively engage in chemical reactions with the physiological environment, thereby promoting tissue integration. For instance, clinically common bone substitute materials—biologically active ceramics or biologically active glass—undergo a series of dissolution-precipitation reactions upon implantation: Ions released from the material surface (such as Na^+^, Ca^2+^) exchange with H^+^ or H_3_O^+^ in bodily fluids, triggering partial dissolution of the silicate network. Subsequently, Ca^2+^ and PO_4_^3−^ precipitate on the surface, forming a thin layer of carbonated hydroxyapatite (carbonated HA). This newly formed HA layer shares a similar chemical composition to natural bone minerals, enabling robust chemical bonding with host tissue. This facilitates seamless integration between the scaffold and newly formed tissue [50,51].

Finally, biodegradability and its degradation products are paramount in scaffold material design. An ideal scaffold should serve as a temporary framework, with its degradation rate precisely matched to the pace of new tissue formation. Should degradation occur too rapidly, mechanical support is lost prematurely; conversely, if degradation proceeds too slowly, it may impede tissue growth and remodeling [43]. More significantly, the degradation products themselves exert a considerable influence on the local microenvironment [52]. For instance, synthetic polyester materials (such as Polylactic Acid (PLA) and Polyglycolic Acid (PGA)), which degrade primarily via hydrolysis, may cause localized accumulation of acidic byproducts (e.g., lactic acid, glycolic acid). This accumulation can lead to a decrease in pH, potentially inducing aseptic inflammation [53]. Conversely, bioactive ceramics like β-Tricalcium Phosphate (β-TCP) release ions such as Ca^2+^ and PO_4_^3−^ during degradation. These are not only safe, metabolizable products but also possess biological activity, acting directly as signaling molecules to stimulate proliferation and differentiation of relevant precursor cells, thereby exhibiting tissue inductivity. Taking β-TCP as an example, it has been extensively utilized in bone replacement materials, with its degradation behavior closely linked to bone tissue regeneration processes: studies demonstrate that the biodegradation of β-TCP is accompanied by osteoblast proliferation, bone matrix formation, and favorable integration between the material and bone tissue [54].

#### 4.1.2. Physical Properties

While biological properties ensure safe implantation and cellular compatibility, the physical properties of a scaffold play a decisive role in determining cell behavior and therapeutic outcomes. The key physical attributes of scaffold materials include structural/morphological and mechanical characteristics.

The scaffold structure and morphology—such as porosity, pore size, and interconnectivity—define the spatial environment available for cell adhesion, proliferation, and nutrient exchange. Studies have shown that scaffolds with high porosity enhance cell infiltration and nutrient diffusion, while an appropriate pore size is critical for efficient cell adhesion, migration, and differentiation. Moreover, the surface topography and roughness of the scaffold significantly influence cell behavior, affecting attachment and lineage commitment.

Mechanical properties are equally crucial. The stiffness, elasticity, compressive strength, and elastic modulus of the scaffold should approximate those of native bone to provide adequate support while delivering suitable mechanical signals to cells. The balance between stiff and soft materials is particularly important: stiffer materials provide superior strength but risk inducing a stress shielding effect, where excessive rigidity reduces mechanical load transfer to surrounding bone, leading to bone resorption. Conversely, overly soft materials may better mimic the elastic environment of natural tissue but may lack sufficient structural stability. Therefore, an optimal scaffold must balance these aspects—offering enough support while maintaining physiological load transfer to promote osteogenesis [55].

### 4.2. Classification of Scaffold Materials

**Figure 2:**
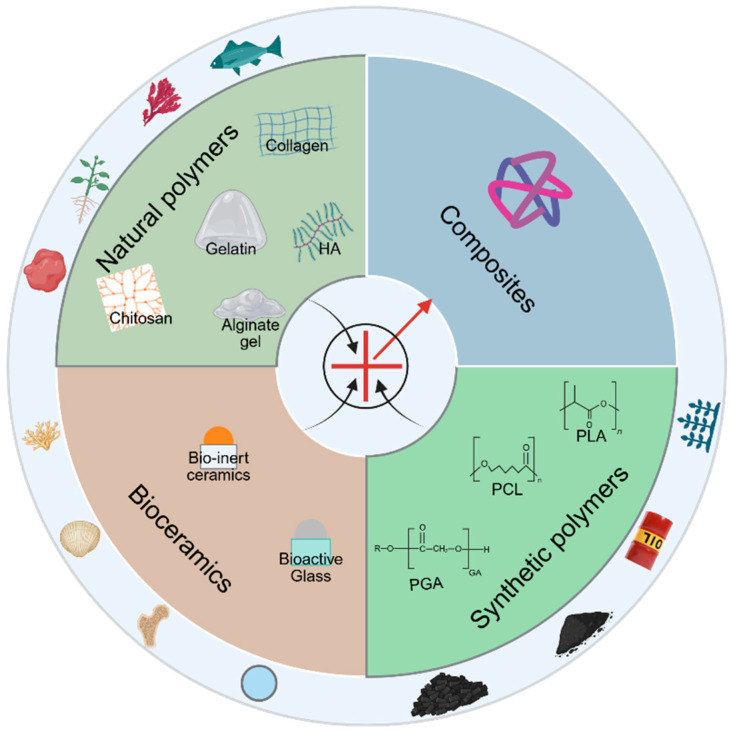
Classification of scaffold materials. The outer ring indicates the material sources, with each sector representing a category and containing the corresponding materials. Created with BioRender.com.

For the different types of bone defects, selecting the appropriate scaffold material is crucial. Based on differences in origin and properties, bone tissue engineering scaffold materials can be primarily categorized into four major types: natural polymers, synthetic polymers, bioceramics, and composite materials (Figure 2).

#### 4.2.1. Natural Polymers

In bone tissue engineering, natural polymers are primarily derived from living organisms, such as animal or plant tissues. Their advantages include good biocompatibility and bioactivity, as they can mimic the extracellular matrix (ECM) and promote cell adhesion and signaling [56,57]. Common materials include collagen, chitosan, gelatin, alginate, and hyaluronic acid [58,59].

Collagen, mainly sourced from animal tissues (e.g., bovine or porcine skin), is a major component of the extracellular matrix and the most common organic template in mineralization [57,60]. Scaffolds derived from collagen not only possess excellent biological properties but also high biodegradability, making them the most prevalent biomaterial in bone tissue engineering [61]. For instance, researcher Hyerim Kim and colleagues used collagen scaffolds encapsulating mesenchymal stem cells (e.g., adipose-derived stem cells, ADSCs) and osteoblasts to promote bone regeneration through paracrine effects [62].

Gelatin is a thermally denatured product of collagen produced through acid/alkali treatment or enzymatic hydrolysis. Compared to collagen, it has better water solubility, gelation ability, and even superior mechanical properties [63]. This substance is often used as a cell carrier, providing a three-dimensional culture environment that promotes cell adhesion, proliferation, and differentiation [64,65].

Chitosan, which is obtained by deacetylation of chitin, is a natural polysaccharide. Its properties—biocompatibility, biodegradability, coadhesiveness, and antibacterial properties—make it an ideal material for bone tissue engineering [66,67]. It promotes osteoblast attachment and bone matrix mineralization, although its osteoinductivity and mechanical strength are relatively low [66,68]. Its performance in promoting bone tissue regeneration is often significantly enhanced by incorporating other inductive molecules or combining it with other biomaterials [69,70].

Besides chitosan, alginate is another commonly used natural polysaccharide for scaffold fabrication, primarily extracted from brown algae or bacteria. It consists of β-D-mannuronic acid (M blocks) and α-L-guluronic acid (G blocks) [71]. In addition to sharing the ability of chitosan to promote osteoblast attachment and bone matrix mineralization [72,73], alginate hydrogels can also slowly release key proteins to guide stem cell differentiation. Studies have shown that *human* umbilical cord mesenchymal stem cells (HUCMSCs) encapsulated in alginate hydrogels can maintain long-term viability and structure, promoting wound healing [74].

Hyaluronic acid (HA) is a glycosaminoglycan naturally found in mammalian connective tissues. It is often used to encapsulate cells whose extracellular matrix is rich in glycosaminoglycans or hyaluronic acid, such as chondrocytes [75,76,77]. In the field of bone regeneration, HA primarily functions by interacting with cells and growth factors involved in bone formation, thereby making it an ideal biomaterial for bone regeneration [78,79]. Furthermore, HA interacts with growth factors like Bone Morphogenetic Proteins (BMPs) and TGF-β, which play crucial roles in bone formation and regeneration [80].

#### 4.2.2. Synthetic Polymers

Although these natural polymers possess good hydrophilicity, biocompatibility, and degradability, the vast majority of them have mechanical strength that is far from sufficient for the requirements of bone regeneration, and their degradation rates are unstable. In contrast, synthetic polymers are primarily obtained through chemical synthesis and offer controllable physicochemical properties, such as molecular weight, degradation rate, and mechanical strength. Common materials mainly include polylactic acid (PLA), polycaprolactone (PCL), and polyglycolic acid (PGA) [81,82,83,84].

Polylactic acid (PLA) is mainly produced from renewable resources like corn starch or sugarcane via fermentation and is one of the biodegradable thermoplastic polyesters [84,85]. PLA is considered an ideal material for bone regeneration due to its good biocompatibility, degradability (2–4 years), and mechanical properties [86]. Its thermoplastic nature makes it suitable for 3D printing technology, enabling the fabrication of bone scaffolds with complex structures that provide a substrate for mesenchymal stem cells (MSCs) to adhere to, guiding cell adhesion, proliferation, and differentiation into osteoblasts.

Polycaprolactone (PCL) is a synthetic aliphatic polyester prepared through the ring-opening polymerization of ε-caprolactone. Compared to PLA, it has a longer degradation time and higher flexibility [87,88,89]. Its slow degradation ensures the overall structural integrity of the scaffold, providing more durable physical support for new bone tissue, making it particularly suitable for repairing bone defects with long regeneration periods (such as large segmental bone defects or bone regeneration in elderly patients) [82,90]. Structurally, PCL scaffolds can be fabricated into nanoscale fibrous structures using a method called electrospinning [91]. These structures promote better attachment and growth of stem cells by mimicking natural tissue.

Polyglycolic acid (PGA) is synthesized from glycolic acid monomers via polycondensation reactions. Compared to PLA and PCL, it exhibits strong hydrophilicity and high support strength, but it has the fastest degradation rate and is often used in combination with various materials [64,65]. The fabrication of poly (lactic-co-glycolic acid) (PLGA) microparticles via the copolymerization of PLA and PCL provides a system for the in situ delivery of rhBMP-2, which exhibits osteogenic and osteoinductive properties [92].

#### 4.2.3. Bioceramics

Bioceramics are inorganic, non-metallic materials that play a key role in the field of orthopedic repair, primarily used for connecting and repairing damaged hard tissues or as filler materials to repair large-sized bone defects. Based on their characteristic biological reactions with host tissues, these materials are mainly divided into two categories: bioinert ceramics and bioactive and biodegradable ceramics. The former maintain stable physicochemical properties within the body, while the latter can actively interact with bone tissue, promoting bone regeneration and healing.

Representative materials for bioinert ceramics mainly include alumina and zirconia. The core advantages of these materials lie in their exceptional stability and mechanical properties. They exhibit high biocompatibility, excellent corrosion and wear resistance, and high compressive strength. Particularly, zirconia is considered one of the strongest implant ceramics due to its high fracture toughness resulting from the “phase transformation toughening” mechanism. However, bioinert ceramics also have inherent drawbacks: the material itself is relatively brittle, especially under tension, and its elastic modulus is much higher than that of natural bone, which somewhat limits their use in applications requiring stress matching with bone [93]. Therefore, they are most commonly used to manufacture permanent implants that require long-term load-bearing and low wear, such as the femoral head and liner in total hip replacements and dental implants [94].

Bioactive and biodegradable ceramics are represented by calcium phosphate (CaP) series ceramics and bioactive glass (BG). They are widely used as bone substitutes, implant coatings, bone cements, and tissue engineering scaffolds [95]. Among them, tricalcium phosphate (TCP) and hydroxyapatite (HAp) are the most common materials in commercial applications [78]. HAp exhibits excellent biocompatibility because its chemical composition is very similar to natural bone, but it degrades slowly and is relatively brittle. TCP, on the other hand, has a faster degradation and absorption rate, able to more quickly make space for new bone tissue, but its mechanical strength is lower than HAp [96,97,98]. To combine the advantages of both, biphasic calcium phosphate (BCP), composed of HAp and TCP, has consequently been developed, achieving controllable degradation rates and excellent osteoconductivity [99].

#### 4.2.4. Composite Materials

Although stem cell scaffolds can be made from natural or synthetic polymers, single-polymer scaffolds still struggle to meet the demands of the physiological process of bone regeneration. Natural polymers possess excellent biocompatibility and bioactivity, enabling them to mimic the extracellular matrix (ECM) and promote cell adhesion and signaling. However, their mechanical strength is insufficient to support load-bearing bone defects, and their degradation rate is difficult to control. In contrast, synthetic polymers offer controllable mechanical strength and degradation properties but suffer from poor bioactivity and weaker cell adhesion capabilities. Therefore, by combining the bioactivity of natural materials with the mechanical support of synthetic materials, preparing composite material scaffolds can better meet the needs of complex bone defect repair and personalized treatment [100]. For example, PCL-HA composite scaffolds loaded with BMSCs increased bone regeneration volume by 40% in a rat calvarial defect model. Kumar Anand et al. found that PCL scaffolds modified with hydroxyapatite (HA) and type I collagen (COL) exhibited stronger osteogenic characteristics and in vitro biocompatibility [64].

### 4.3. Functionalization Strategies of Stem Cell Scaffolds

Although traditional biomaterial scaffolds, such as PLGA, PCL, or ceramic materials, possess basic biocompatibility and structural support functions, their inherent “passivity” limits their application potential in the field of bone repair. While these materials are osteoconductive, they generally lack osteoinductivity; they cannot actively guide stem cells toward the osteogenic lineage, nor can they recreate the complex spatiotemporal signaling environment of natural bone healing [101]. Therefore, biofunctionalization has become a core concept in the design of a new generation of bone repair scaffolds. Biofunctionalization refers to the integration of specific bioactive signal molecules or structures into the scaffold material through physical, chemical, or biological methods, transforming it from a passive physical template into a “dynamically regulatory” scaffold capable of actively controlling cell behavior and guiding tissue regeneration. Its core objective is to endow the scaffold with osteoinductivity, efficiently driving the osteogenic differentiation of stem cells and new bone formation by mimicking the composition and function of the natural extracellular matrix (ECM), or by simulating the signal cascades present during bone healing.

#### 4.3.1. Scaffold Surface Functionalization Strategies

Scaffold surface functionalization is a key strategy in the field of bone regeneration, aimed at optimizing the biocompatibility, bioactivity, and integration ability with host tissues by modifying the surface properties of the scaffold material, thereby promoting the repair and regeneration of bone defects [5,102]. These strategies encompass physical, chemical, and bioactive molecule levels, collectively enhancing the scaffold’s osteointegration and regeneration potential [5,103].

Peptide and Nucleic Acid Aptamer Modification: By covalently linking bioactive peptides or nucleic acid aptamers to the scaffold surface, the recruitment and differentiation of stem cells can be specifically guided [104].

① Peptides: Valued for their unique bioactivity, high specificity, and tunability, peptides often serve as sources for drug lead compounds and have become core tools in the functionalization of stem cell scaffolds. Common functional peptides include the Arginine-Glycine-Aspartic acid (RGD) peptide, Osteogenic Growth Peptide (OGP), and Cell-Penetrating Peptides (CPPs).

The RGD peptide, as the most classical adhesion sequence in extracellular matrix (ECM) proteins, can specifically bind to integrin receptors on the cell surface [105,106]. Chemically conjugating the RGD peptide to the surface of scaffold materials (e.g., polycaprolactone, PCL) can significantly improve the adhesion, proliferation, and osteogenic differentiation of mesenchymal stem cells (MSCs) [102,105,107,108]. This modification strategy effectively mimics the microenvironment of natural bone tissue, providing “anchoring points” for cells, thereby guiding cell behavior and ultimately accelerating the bone regeneration process.

**Figure 3:**
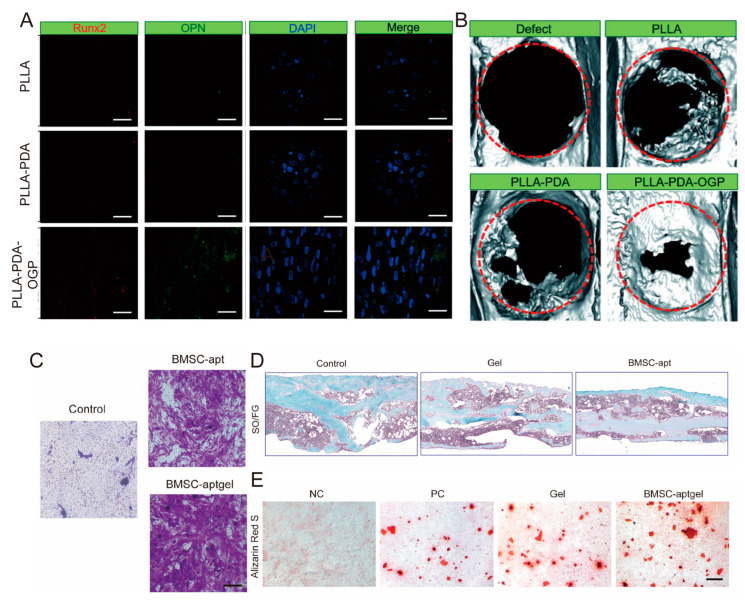
Research Findings Related to Peptide Segment and Nucleic Acid Aptamer Modifications. (**A**) Double staining with Runx2 and OPN immunofluorescence in hMSCs cultured on skeletal tissue for 14 days [112]. (**B**) Reconstructed micro-CT images of bone samples cultured on skeletal tissue [112]. The dashed red circles indicate the original extent of the critical-sized calvarial defect. (**C**) Representative images of crystal violet-stained migrating cells [115]. (**D**) Representative images of SO/FG staining after various treatments over 3 weeks. Scale bar: 250 μm, n = 5 per group [115]. (**E**) Representative images of alizarin red S staining for mineral deposits in BMSCs in bone-like medium. Scale bar: 200 μm, n = 3 per group [115].

Osteogenic Growth Peptide (OGP) is a sequence composed of 14 amino acids (NH_2_-ALKRQGRTLYGFGG-OH), and its C-terminal pentapeptide (YGFGG), namely OGP (10–14), is considered its primary physiologically active form. Numerous studies have confirmed that OGP and its active fragments can effectively stimulate the proliferation, differentiation, alkaline phosphatase (ALP) activity, and matrix mineralization of osteoblastic lineage cells, and effectively increase bone formation and trabecular density in vivo. Based on its excellent bioactivity, loading OGP onto biomaterial scaffolds has become an ideal surface functionalization strategy to enhance the osteoinductive capacity of materials [109,110,111]. For instance, Yong Liu et al. used a polydopamine (PDA) coating to immobilize OGP on a PLLA scaffold (PLLA-PDA-OGP). This scaffold exhibited excellent cytocompatibility and significantly enhanced the adhesion, proliferation, and osteogenic differentiation of *human* mesenchymal stem cells (hMSCs) (Figure 3A). Furthermore, in vivo studies using a rat calvarial defect model confirmed that the PLLA-PDA-OGP scaffold significantly promoted bone regeneration compared to the unmodified PLLA scaffold [112] (Figure 3B).

Although Cell-Penetrating Peptides (CPPs) are primarily used in drug delivery systems, their potential application at the cell/scaffold interface is gradually being explored [113]. For example, CPPs can be utilized to efficiently deliver osteoinductive factors to deep regions within the scaffold or directly into adhered cells, thereby exerting more precise and efficient regulatory effects within the bone regeneration microenvironment [114].

② Nucleic Acid Aptamers: Nucleic acid aptamers, also known as “chemical antibodies,” are single-stranded DNA or RNA molecules that can bind target molecules with high affinity and specificity. Due to their unique molecular recognition capabilities, low immunogenicity, and ease of chemical modification, they show great potential in the functional modification of bone regeneration scaffolds and have become a research hotspot in bone tissue engineering. These characteristics give them significant advantages in precisely regulating stem cell behavior and enhancing bone regeneration outcomes. A key application of nucleic acid aptamers in bone regeneration is the precise recruitment of endogenous stem cells. Jiang-Shan Gong et al. developed a PEG hydrogel loaded with a specific bone marrow-derived mesenchymal stem cell (BMSC) aptamer (BMSC-aptgel). Both in vitro and in vivo experiments demonstrated that this functionalized hydrogel significantly increased the recruitment and migration of BMSCs, effectively promoting fracture healing [115] (Figure 3C–E). Beyond recruiting cells, nucleic acid aptamers can also serve as efficient molecular recognition tools for the delivery or anchoring of osteogenic growth factors. For instance, one study identified a novel DNA aptamer, BA1, that specifically binds to recombinant *human* Bone Morphogenetic Protein-2 (rhBMP-2). The researchers functionalized a type I collagen scaffold with this aptamer, utilizing its specific binding capability to capture and immobilize rhBMP-2. The results showed that this aptamer-functionalized scaffold significantly enhanced the osteoinductive activity of rhBMP-2 in vitro [116]. Furthermore, nucleic acid aptamers can be used for the targeted modification of other therapeutic carriers, such as exosomes, to achieve precise delivery. One study utilized a phosphatidylserine (PS)-targeting aptamer to combine reparative Schwann cell-derived exosomes with a biomimetic periosteum, which promoted the regeneration of damaged peripheral nerves and subsequently enhanced bone regeneration effects [104].

Polydopamine Coating: Polydopamine (PDA) is a biomimetic coating formed by the polymerization of dopamine (DA) in a weak alkaline solution. Due to its excellent adhesion and biocompatibility, it has become a widely used surface modifier, showing great potential particularly in the field of bone regeneration [117]. The primary roles of PDA coating are twofold: first, to directly improve the biological properties of the material surface, and second, to serve as a functional “anchoring layer” for immobilizing bioactive molecules [118]. First, the PDA coating itself can actively regulate the bone regeneration microenvironment. Its structure contains a large number of hydrophilic groups, such as amino and hydroxyl groups, which can significantly improve the hydrophilicity of hydrophobic material surfaces like polylactic acid (PLLA) or titanium alloys, thereby promoting cell attachment. Studies have confirmed that the nanoscale rough surface formed by PDA can enhance the adhesion, proliferation, and aggregation of osteoblasts. Simultaneously, PDA is an effective mineralization inducer; its unique catechol structure can chelate calcium ions, providing a platform for the nucleation and crystallization of hydroxyapatite (HAp), the main inorganic component of bone, thus accelerating the mineralization process [119]. Beyond the chelation of calcium, the catechol moieties of PDA exhibit a broad capacity to bind a wide spectrum of divalent and trivalent metal ions. This includes magnesium (Mg^2+^), as well as zinc (Zn^2+^) and copper (Cu^2+^), which are further discussed for their osteogenic properties in Section 4.3.3 [120]. The primary mechanism for this immobilization is the formation of coordination bonds. Consequently, the binding affinity is not uniform and varies based on the distinct physicochemical properties of each ion, such as its charge, ionic radius, and Lewis acidity [121]. This differential binding affinity is a critical factor that subsequently governs the ions’ release kinetics and resultant bioactivity. Furthermore, the PDA coating itself is non-cytotoxic and can enhance the overall biocompatibility of implants by modulating cell differentiation and inflammatory responses. For example, constructing a PDA coating on the surface of poly-L-lactic acid (PLLA) material not only enhances its immunogenicity and biocompatibility but also effectively reduces the hemotoxicity of CdSe quantum dots loaded on its surface [122]. Second, and more importantly, the powerful adhesive capability of PDA allows it to serve as a universal intermediate linking layer, firmly fixing various functional drugs or biomolecules onto the implant surface. In this way, a variety of active substances such as Bone Morphogenetic Protein-2 (BMP-2), the Arginine-Glycine-Aspartic acid (RGD) short peptide, strontium ions (Sr), and even antibiotics can be uniformly anchored to the scaffold. This immobilization not only prevents the burst release of drugs but also enables their long-term, stable release at the lesion site, thereby more effectively guiding cell behavior and constructing a microenvironment conducive to bone repair. For instance, introducing a PDA coating on a hydroxyapatite (HA)-modified titanium alloy (Ti6Al4V) surface can effectively anchor growth factors like BMP-2, achieving uniform distribution and sustained release in vivo [123].

Chemical Modification: This generally refers to altering the surface chemical properties of the scaffold through treatment to enhance cell adhesion and osteogenic differentiation. For example, Boda Ying et al. developed a method for rapid, bulk surface modification of 3D-printed polyetherimide (PEI) high-strength polymer scaffolds through sulfonation or plasma treatment, enhancing their bioactivity and improving implantation efficiency [124].

#### 4.3.2. Growth Factor Loading Functionalization Strategy

Integrating growth factors into biological scaffolds is a crucial functionalization strategy aimed at significantly enhancing the osteoinductive properties of the material, thereby promoting tissue repair and regeneration. Growth factors widely studied in the field currently include Bone Morphogenetic Proteins (BMPs), Vascular Endothelial Growth Factor (VEGF), Fibroblast Growth Factors (FGFs), and the Transforming Growth Factor-β (TGF-β) family [125,126].

Bone regeneration relies on the close coupling of angiogenesis (new blood vessel formation) and osteogenesis (new bone formation). VEGF is a key factor initiating angiogenesis, providing nutrients and oxygen to the repair site; whereas BMPs (such as BMP-2 and the more potent BMP-9) are among the strongest osteoinductive factors [127,128,129]. To mimic the natural healing sequence of “vascularization first, osteogenesis later,” researchers have developed intelligent delivery systems [125]. For instance, loading VEGF into a heparinized gelatin-hydroxyapatite-tricalcium phosphate (Hep-Gel-HA-TCP) composite scaffold not only significantly increased ALP activity in cells, promoted mineralized nodule formation, and upregulated the gene and protein expression of various osteogenic markers but also markedly enhanced the migration, invasion, and tube formation abilities of vascular endothelial cells [130]. Furthermore, Bojun Cao et al. developed an intelligent composite hydrogel responsive to the acidic microenvironment present in the early stages of bone injury. This system chemically tethers VEGF to the hydrogel for rapid release, promoting early vascularization [131]; simultaneously, BMP-2 is encapsulated in mesoporous silica nanoparticles (MSNs) for sustained slow release, supporting subsequent bone formation. This temporal delivery strategy effectively recapitulates the physiological repair process.

In addition to BMPs and VEGF, FGFs and TGF-β also participate in the regulation of bone tissue repair and remodeling through unique signaling pathways. FGFs primarily regulate cell behavior by binding to Fibroblast Growth Factor Receptors (FGFRs) on the cell surface, activating downstream signaling pathways such as MAPK/ERK, PI3K/AKT, and PLCγ [132]. For example, loading FGF-2 onto multi-layered poly (L-lactic acid) (PLLA) nanofilms enabled controlled release, leading to sustained activation of the FGF signaling pathway and significantly promoting the regeneration of large segmental defects in mouse femurs [133]. On the other hand, the role of TGF-β in bone regeneration exhibits high complexity and context-dependency, with its effects depending on concentration, cell type, and the local microenvironment. It primarily exerts its pleiotropic regulatory functions by binding to TGF-β receptors on the cell surface and activating the Smad signaling pathway [134].

#### 4.3.3. Bioactive Ion Doping Functionalization Strategy

A stable ionic environment is a crucial condition for maintaining bone growth and development, playing a key role in bone metabolism [135,136]. Therefore, in the field of bone regeneration, integrating these bioactive inorganic ions into biological scaffolds is a key strategy for enhancing their functionality. Currently, the most extensively studied bioactive ions mainly include zinc ions (Zn^2+^), magnesium ions (Mg^2+^), strontium ions (Sr^2+^), copper ions (Cu^2+^), calcium ions (Ca^2+^), and phosphate ions (PO_4_^3−^). These ions exert their unique biological effects through diverse mechanistic networks. For example: magnesium ions (Mg^2+^) can efficiently promote osteogenic differentiation [137]; strontium ions (Sr^2+^) can combat osteoporosis by inhibiting osteoclast activity [138]; zinc ions (Zn^2+^) provide crucial antibacterial capability to address implant-related infections [139]; and copper ions (Cu^2+^) play a central role in driving vascularized bone regeneration. Furthermore, ions such as lithium ions (Li^+^), silicon ions (Si^4+^), and silver ions (Ag^+^) also show great potential in specific applications (e.g., osteochondral repair, antibacterial coatings) [140,141,142,143]. They primarily function by regulating key cell signaling pathways (such as Wnt, MAPK, HIF-1α, etc.), influencing the expression of osteogenesis-related genes, optimizing the composition and mineralization of the extracellular matrix, and promoting local angiogenesis, thereby precisely controlling the proliferation and osteogenic differentiation of stem cells, significantly promoting bone tissue formation, and accelerating the defect repair process (Table 1). Consequently, when designing and developing the next generation of stem cell scaffolds, how to precisely control the release kinetics (e.g., release rate and local concentration) of these ions and their interaction with the scaffold substrate has become a core challenge for achieving efficient bone regeneration. Future research will continue to focus on exploring the application potential of novel bioactive ions and dedicating efforts to utilizing the synergistic effects of multiple ions to construct multifunctional biomaterial platforms, aiming for a synergistic therapeutic effect where “1 + 1 > 2” and expecting superior clinical application prospects.

#### 4.3.4. Extracellular Vesicle (EV) Functionalization Strategy

Extracellular vesicles (EVs)—particularly exosomes secreted by mesenchymal stem cells (MSCs)—represent a promising cell-free therapeutic modality capable of transferring bioactive molecules to target cells and modulating the regenerative microenvironment. Integrating EVs with biocompatible scaffolds enhances their retention, stability, and osteoinductive efficacy. For instance, aptamer-engineered exosomes immobilized on a biomimetic periosteum were shown to promote angiogenesis and bone regeneration by activating the JNK3/MAPK pathway in injured neural tissues [104]. Similarly, Li Wang et al. demonstrated that hydrogels loaded with osteoinductive dental pulp stem cell–derived EVs (Ost-EVs) significantly enhanced osteogenesis in preclinical bone defect models [20]. These findings underscore that EV-functionalized scaffolds not only deliver regenerative cues but also serve as dynamic microenvironments capable of coordinating angiogenesis, osteogenesis, and immune modulation—offering a new frontier for next-generation bone tissue engineering.

Moreover, the unique properties of matrix vesicles (MVs) offer novel avenues for functionalizing biomaterials. As a distinct subset of EVs, MVs have been demonstrated to specifically initiate bone mineralization, primarily owing to their highly specialized innate functionality [164,165]. On the one hand, MVs undergo complex biogenesis processes; studies indicate they can form either through direct budding from the plasma membrane (a budding-like process) or via multivesicular bodies (a secretory vesicle-like process) [166,167]. On the other hand, matrix vesicles possess critical targeted anchoring capabilities. Their surface enrichment with components such as annexins and tissue-nonspecific alkaline phosphatase (TNAP/ALP) enables them to adhere to or bind with collagen [168,169]. This structural property enables MVs to precisely localize and initiate the mineralization process. Consequently, in developing functionalized biomaterials, whether directly utilizing MVs or mimicking their characteristics (such as anchoring capacity and enzymatic activity) through biomimetic approaches, presents highly promising strategies for achieving targeted, efficient bone tissue engineering.

#### 4.3.5. Microenvironment Regulation-Based Functionalization Strategy

Bone regeneration is a complex biological process influenced by multiple factors in the local microenvironment, including mechanical stimuli, oxygen supply, inflammatory responses, and cell–cell interactions [19].

Mechanical Microenvironment: The mechanical properties of the scaffold, such as pore structure and stiffness, are crucial for the osteogenic differentiation of stem cells [170]. 3D printing technology allows precise control over the scaffold’s geometry and pore structure, thereby optimizing mechanical support and cell infiltration [171]. Studies indicate that scaffolds with appropriate pore size and interconnected structures promote cell migration, nutrient transport, and neovascularization, providing favorable conditions for bone regeneration. For instance, scaffolds fabricated via 3D printing with specific pore sizes (e.g., 300–500 μm) have been proven effective in promoting the osteogenic differentiation of bone marrow mesenchymal stem cells (BMSCs) and in vivo bone formation [172]. The stiffness of the scaffold material also influences stem cell fate; moderate stiffness promotes osteogenic differentiation of MSCs, while excessively high or low stiffness may inhibit osteogenesis or induce alternative differentiation pathways. Beyond static mechanical properties, dynamic mechanical stimulation (such as cyclic compression, tension, or shear stress) is also recognized as an important factor promoting bone regeneration. Bioreactor systems can simulate the complex mechanical environment in vivo, applying specific mechanical stimuli to cell-laden scaffolds, thereby enhancing cell proliferation and osteogenic differentiation [102]. For example, some studies have explored methods for transmitting mechanical signals using hydrogels or piezoelectric materials (such as BaTiO3-doped PCL). These materials generate electrical signals upon mechanical stress, which can modulate cell behavior and promote angiogenesis. Furthermore, the piezoelectric effect might synergistically promote angiogenesis and osteogenesis through mechanisms such as regulating the opening of intracellular calcium channels and activating signaling pathways (e.g., PI3K/Akt, MAPK) [173,174].

Oxygen Microenvironment: Hypoxia can impair bone tissue healing, making the construction of an oxygen-rich microenvironment crucial for bone regeneration [10]. Researchers often achieve sustained oxygen supply within scaffolds by loading oxygen-producing nanoparticles or simulating photosynthetic systems, thereby promoting osteogenesis and angiogenesis [126]. Additionally, the aforementioned loading of VEGF growth factors can actively induce the formation of new blood vessels, thereby alleviating local hypoxia and promoting cell survival and vascularization. For instance, Xiao Sheng et al. introduced sodium alginate and VEGF onto functionalized mineralized collagen (FMC), finding that it enabled sustained release of the growth factor and more effectively promoted angiogenesis and osseointegration [174,175].

Immune Microenvironment: The immunogenicity of scaffold materials and their interaction with host immune cells are key factors determining the success of bone regeneration [176]. The polarization of macrophages plays a significant role in bone regeneration and repair, making it a key target for strategies aimed at regulating the immune microenvironment at bone defect sites. Combining bioceramic materials to induce macrophage polarization is currently one of the effective strategies. For example, CSC-DBM bioceramics, based on calcium silicate (CS), calcium sulfate hemihydrate (CSH), and decellularized bone matrix (DBM), were designed for immunomodulation, vascularization, and osteogenesis. They demonstrated excellent biomineralization, induction of M2 macrophage polarization, and promotion of angiogenesis and osteogenic differentiation in calvarial defect repair [177]. Other methods include the aforementioned strontium ion doping, cytokine and growth factor loading/delivery, and exosome delivery strategies, which can suppress harmful inflammation and promote M2 macrophage polarization, thereby creating favorable conditions for angiogenesis and osteogenesis [153].

## 5. Prospects of Biofunctionalization of Stem Cell Scaffold Based on Bibliometric Study and Clinical Trials

Publications are widely recognized as a key indicator of research trends and contributions, serving as a vital element in scientific research. Consequently, bibliometric analysis is extensively utilized to analyze large volumes of research data and identify emerging patterns. Through citation networks, it becomes possible to track the evolution of research papers, predict emerging research areas, and assess critical challenges within specific fields. Bibliometric tools such as CiteSpace 6.3.R1, VOSviewer 1.6.14, and R packages 4.5.0 are used to visualize the analysis of medical literature within specific fields [178,179]; however, bibliometric studies on the application of functional stem cell constructs in bone formation and regeneration have not been sufficiently published to date. Furthermore, simulation analyses regarding the use of functional stem cell constructs in these processes are also lacking. Therefore, this section addresses this knowledge gap using bibliometrics, comprehensively analyzing the literature on microcrystals over the past decade (2014–2024), visualizing the data to identify key features, and predicting future research trends.

### 5.1. Global Literature Trend

The Web of Science Core Collection (WoSCC) database from Clarivate Analytics was used to obtain bibliographic data for this study. It is considered a comprehensive and reliable source of information. By establishing a specific search strategy, this study systematically retrieved research data on stem cell scaffold for osteogenesis and bone regeneration from 2014 to 2024 and back to 18 August 2025. The search strategy used in this study was as follows: topic = stem cells or mesenchymal stem cells or MSCs or MSCs AND topic = osteogenesis or bone regeneration AND topic = scaffold AND topic = functionalization or functionalized AND publication year = (2014 to 2024) AND document type = (article or review) AND language = (English). Based on the search criteria for the period between 2014 and 2024, a total of 640 relevant articles were collected. After excluding articles from proceeding papers (4 articles), book chapters (2 articles), corrections (2 articles), early access publications (2 articles), and non-English publications, 630 articles were identified.

**Figure 4:**
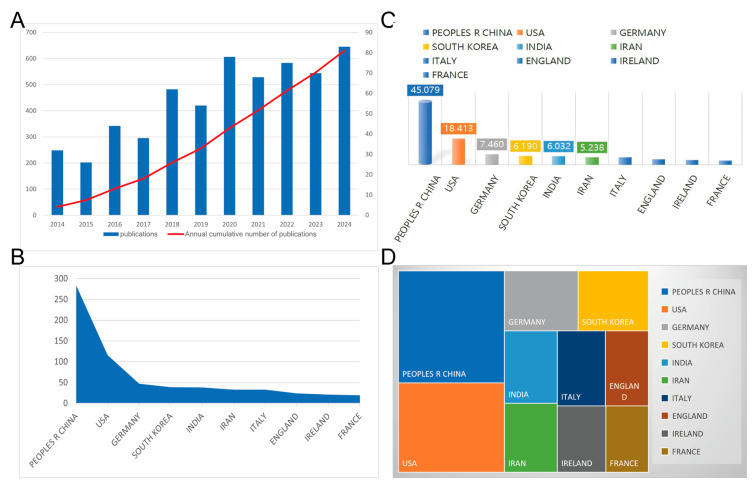
Global Literature Trend. (**A**) Annual publication volume and cumulative publication volume globally in this field. (**B**) Top 10 countries or regions in terms of total citations in the field. (**C**) The percentage of publications by country in this field. (**D**) Top 10 countries and regions in terms of H-index in the field of mesenchymal stem cell therapy for bone regeneration.

As shown in Figure 4A, since 2024, the annual number of papers published has consistently exceeded 30; after reaching a peak of about 80 in 2020, the number remained stable above 80 for the next four years. The number of annual papers has exceeded 100 every two years for the past 10 years—this data reflects the sustained and significant interest of researchers in the application of stem cell structures in bone formation and regeneration. Figure 4B shows that the largest contributions to publications on this topic over the past decade have come from China, followed by the United States, Germany, and South Korea. However, Figure 4C shows that major contributions are still concentrated in China and the United States, with China accounting for nearly half of the total publications on this topic over the past decade, and the United States accounting for nearly one-fifth. Other countries have a share of less than 10%. Figure 4D also shows the H-index for the top 10 countries in this field. China leads the pack with the highest H-index (59), followed by the United States (47), Germany (22), and South Korea (21). We attribute China’s high H-index not only to the large number of papers it has published over the past decade but also to the fact that Chinese papers received 11,843 citations, compared to only 6350 citations for the United States.

### 5.2. Bibliometric Analysis of Cooperation Among Authors, Countries, and Institutions

**Figure 5:**
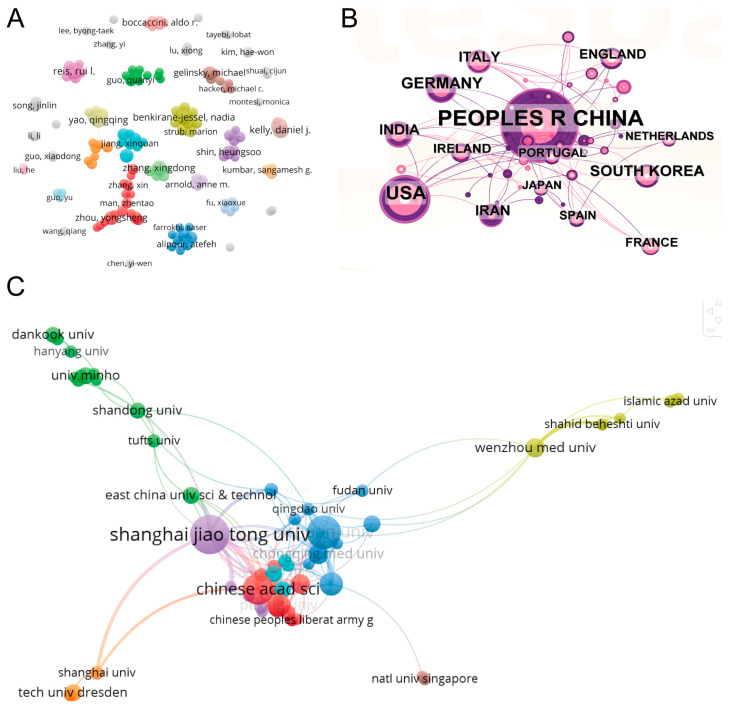
Bibliometric Analysis of Cooperation Among Authors, Countries, and Institutions. (**A**) Mapping of the identified authors in this field based on VOSviewer. (**B**) National collaboration analysis and (**C**) Institutional collaboration analysis in this field.

We extracted comprehensive bibliographic details from 630 relevant publications from the Web of Science Core Collection (WoSCC), including year of publication, title, author names, nationality, affiliated institution/association, abstract, keywords, and journal title. We converted this data into a downloadable format. We then imported the dataset into CiteSpace and VOSviewer to systematically analyze the collaboration networks between authors, countries, and institutions in this field of research (Figure 5).

The 630 publications included a total of 3907 authors. By applying two filtering criteria ―” minimum number of documents an author—3” and “minimum number of citations an author ≥10”—we identified 136 key authors who met these restrictions and presented their collaboration networks (Figure 5A). The nodes in the network showed clear clustering characteristics—different colors marked separate collaboration groups, with denser connections between nodes of the same color indicating stronger academic ties. Particularly prominent are Benkirane-Jessel, Nadia (total connection strength 33), Alipour, Atefeh (27), Hosseini, Saadi (27), Shahsavarani, Hosein (27), and Bornert, Fabien (26), who are among the top five in terms of connection strength. It is reasonable to assume that these key authors are important leaders in the field and that their research has a significant impact on academia.

Figure 5B shows the national cooperation network in this field, which was created using the CiteSpace tool (parameter settings: Slice Length = 4, g-index(k = 20), LRF = 2.5, L/N = 10, LBY = 5, e = 1.0). This visualization clearly shows both the geographical differences in global research capabilities and the intensity of collaborative ties. The data show a clear inequality in the distribution of open access publications among countries on this topic. It should be noted that scientists from China collaborate particularly closely with their colleagues from the United States, South Korea, it is worth noting that the ten countries with the highest number of publications (including China, the United States, South Korea, and Germany) are also the main participants in international collaboration. Thanks to frequent academic exchanges, these countries have created a global knowledge network in this field, underlining their dominant position in the world. Thanks to frequent academic exchanges, these countries have created a global knowledge network in this field, underlining their dominant position in global scientific research.

Figure 5C shows a visualization design based on nodes and connection lines that illustrates the topology of collaboration at the institutional level. The analysis identified key research institutions in this field, including Shanghai Jiao Tong University, the Chinese Academy of Sciences, Huazhong University of Science and Technology, Sichuan University, Trinity College Dublin, Peking University, Wenzhou Medical University, and Qingdao University. Furthermore, we can see that these institutions are divided into eight separate groups (indicated by different colors) according to the closeness of their cooperation with each other. Within each group, the connections between institutions are very tight, reflecting a highly synergistic model of knowledge production. A striking example is the red group, consisting of Beijing University of Chemical Technology, Chinese Academy of Sciences, Harvard Medical School, Nankai University, and Peking University. Here, collaborative activities among these institutions are much more frequent than in other color-coded groups (green group: Hunan University, blue group: Sichuan University, yellow group: Iran Pasteur Institute, etc.). This indicates that institutions within the same color group form a relatively stable academic community in which specific research areas are deeply developed through sustained cooperation. In contrast, intergroup cooperation is more strongly dependent on the existence of development paths and barriers.

### 5.3. Clinical Trials of Biofunctionalized Scaffolds for Bone Regeneration

The combination of stem cells with biological scaffolds represents a major direction in the field of tissue engineering. These constructs are primarily used for conditions requiring structural repair and bone regeneration, such as large craniofacial defects [180], femoral head necrosis [181], and nonunion of long bones [182]. When evaluating clinical studies, in addition to the basic assessments of efficacy and safety, seeding efficiency and cell viability are critical indicators of functionalized scaffolds [183]. To ensure osteogenic bioactivity, most implanted cells are mesenchymal stem cells (MSCs) with osteogenic potential, including bone marrow–derived MSCs (BM-MSCs) and adipose-derived stem cells (ASCs) [181,184].

In 2011, Thesleff et al. [185] conducted a clinical study in which four patients with large cranial defects underwent reconstruction using autologous ASCs combined with β-tricalcium phosphate (β-TCP) granules. Computed tomography (CT) scans demonstrated satisfactory ossification, with Hounsfield unit (HU) values of the grafts gradually increasing to levels comparable to native bone, and no postoperative complications were observed [185]. Seven years later, Sponer et al. reported a prospective controlled trial evaluating autologous expanded BM-MSCs combined with β-TCP scaffolds (Group A) for femoral defect repair, in comparison with β-TCP alone (Group B) or allogeneic bone grafts (Group C, control). The results revealed that bone healing in Group A (MSC–scaffold) was comparable to Group C (allograft) but significantly superior to Group B (scaffold alone) [182]. Additionally, Zhuang et al. developed an intraoperative device known as the stem cell screen–enrich–combine(-biomaterials) circulating system (SECCS), which can rapidly process autologous bone marrow within 10–15 min. This system screened and enriched over 85% of MSCs and simultaneously seeded them onto β-TCP scaffolds without any ex vivo cell culture. All 42 patients achieved satisfactory bone healing, marking a significant breakthrough in the field [186].

However, due to both technical challenges inherent to stem cell–based scaffolds and ethical constraints associated with stem cell use, clinical trials of functionalized scaffolds remain largely in the early stages, focusing mainly on feasibility and safety evaluations [187]. Beyond conventional clinical ethics, particular attention must be given to the ethical implications introduced by the technological uncertainty of rapidly evolving biomaterials. These include transparency in informed consent, unknown safety and efficacy, potentially misleading expectations for patients, and ethical issues related to resource allocation. Furthermore, stem cell therapies raise additional concerns such as ethical issues regarding cell sourcing, commercialization and market-driven motivations, as well as potential side effects and risks—including tumorigenicity and chromosomal aberrations associated with ex vivo cell expansion. Compounding these challenges are regulatory delays and inconsistent review standards across countries. In the future, more randomized controlled trials (RCTs) are urgently needed to further validate the safety and efficacy of stem cell–functionalized scaffolds and to accelerate their clinical translation in bone regeneration therapies.

## 6. Conclusions

The comprehensive development of materials science, tissue engineering, and regenerative medicine, combined with an in-depth understanding of the complex microenvironment of bone regeneration, has led to the identification of biofunctional scaffolds as an innovative tissue engineering strategy. Today, there is currently a consensus that these scaffolds have significant clinical potential. The continuous innovation in functionalization strategies thus serves as the cornerstone for developing a new generation of regenerative scaffolds that integrate both bioactivity and sophisticated functionalities.

The design philosophy of these scaffolds has undergone a fundamental transformation, from inert structures that only provide passive support to dynamic bioreactors that actively control the fate of cells and remodeling processes. Their functional capabilities are realized through multiple avenues: For instance, surface chemical modifications can recruit endogenous stem cells to enhance innate repair mechanisms; integrated smart-responsive modules can perceive dynamic microenvironmental changes (e.g., inflammation, hypoxia) and thereby enable the spatiotemporally controlled release of therapeutic molecules; additionally, these scaffolds act as efficient delivery vehicles for bioactive ions, cytokines, or extracellular vesicles, thereby synergistically orchestrating vascularization and osteogenesis.

The bibliometric analysis presented in this article underscores the field’s rapid growth trajectory. The data indicate that important areas of research stimulating future development include, among others, 3D bioprinting, the immune microenvironment, controlled drug release, vascularization strategies, and the use of extracellular vesicles.

However, it is a big challenge to turn these sophisticated, functional scaffolds, which have shown promising preclinical results, into clinical applications. The primary translational bottlenecks encompass the following: First, the difficulty in achieving high-fidelity simulation of the spatiotemporal dynamics of multiple signaling cues present in natural bone healing. Second, ensuring the long-term biocompatibility and safety of composite biomaterials and their metabolic byproducts. And third, navigating the increasingly rigorous regulatory approval pathways for medical products.

In conclusion, despite these formidable challenges, the outlook remains promising. Should these advanced biofunctionalized scaffold technologies successfully overcome the translational barriers, they are poised to revolutionize the treatment paradigm for challenging orthopedic conditions, such as critical-sized bone defects and osteoporotic fractures. Such innovative therapies have the potential to fundamentally improve clinical outcomes, reduce healthcare burdens, and enhance patient well-being—their profound clinical and societal value is undeniable.

## Figures and Tables

**Table 1:** The Functions and Applications of Bioactive Ions in Bone Repair Materials.

Classification	Biological Functions	Applications
Mg^2+^	Activating Wnt/PI3K/MAPK pathways [144,145]. Upregulating angiogenic factors like VEGF [146,147,148]. Inducing M2 macrophage polarization [149].	Enhances osteogenesis in various bone repair materials.
Sr^2+^	Inhibiting osteoclastogenesis via OPG/RANKL pathway [138,150]. Promoting osteogenesis via Wnt/ERK pathways [151,152] Inducing M2 macrophage polarization [153].	First-line choice for treating osteoporotic bone defects.
Zn^2+^	Generating ROS for antibacterial effects [139]. Activating MAPK/PI3K pathways to promote osteogenesis and angiogenesis [154]. Inducing M2 polarization for immunomodulation [155].	Used for infected bone defects and anti-infective implants.
Cu^2+^	Stabilizing HIF-1α and activating ERK pathways to promote angiogenesis [156,157]. Catalytically generating ROS for antibacterial effects [158]. Activating ERK/Akt pathways to promote osteogenesis [159].	For large/poorly vascularized or infected bone defects.
Ca^2+^ and PO_4_^3−^	Activating Wnt/β-catenin pathway to promote stem cell migration and osteogenic differentiation [143] Enhancing mineralization through Wnt/β-catenin activation [160]	Repairs large or hypovascular bone defects.
Li^+^	Activating the Wnt/β-catenin signaling pathway to promote osteogenic differentiation [161,162].	Used to promote bone regeneration, particularly showing potential in complex osteochondral interface repair.
Other Bioactive Ions	Ag^+^: exerting dual antibacterial/anti-inflammatory effects [142]. F^−^: enhancing hydroxyapatite crystallinity and stability [163].	Used for antibacterial coatings or dental materials.

## Data Availability

The raw data supporting the conclusions of this article will be made available by the authors on request.
